# Transcriptional Organization of the Stability Module of Broad-Host-Range Plasmid RA3, from the IncU Group

**DOI:** 10.1128/AEM.00847-20

**Published:** 2020-08-03

**Authors:** Ewa Lewicka, Patrycja Dolowy, Jolanta Godziszewska, Emilia Litwin, Marta Ludwiczak, Grazyna Jagura-Burdzy

**Affiliations:** a Institute of Biochemistry and Biophysics, Polish Academy of Sciences, Warsaw, Poland; University of Tokyo

**Keywords:** RNAP read-through, broad-host-range plasmid, gene expression, stability functions, transcript dosage

## Abstract

The broad-host-range (BHR) conjugative plasmids have developed diverse adaptive mechanisms defining the range of their promiscuity. The BHR conjugative RA3 plasmid, the archetype of the IncU group, can transfer between, replicate in, and be maintained in representatives of *Alpha*-, *Beta*-, and *Gammaproteobacteria*. Its stability module encompasses ten open reading frames (ORFs) apparently organized into five operons, all transcribed in the same direction from several strong promoters that are tightly regulated either by autorepressors or by global plasmid-encoded regulators. In this paper, we demonstrate that owing to an efficient RNA polymerase (RNAP) read-through, the transcription from the first promoter, *orf02p*, may continue through the whole module. Moreover, an analysis of mRNA produced from the wild-type (WT) stability module and its deletion variants deprived of particular internal transcription initiation sites reveals that in fact each operon may be transcribed from any upstream promoter, giving rise to multicistronic transcripts of variable length and creating an additional level of gene expression control by transcript dosage adjustment. The gene expression patterns differ among various hosts, indicating that promoter recognition, regulation, and the RNAP read-through mechanisms are modulated in a species-specific manner.

**IMPORTANCE** The efficiently disseminating conjugative or mobilizable BHR plasmids play key roles in the horizontal spread of genetic information between closely related and phylogenetically distant species, which can be harmful from the medical, veterinary, or industrial point of view. Understanding the mechanisms determining the plasmid’s ability to function in diverse hosts is essential to help limit the spread of undesirable plasmid-encoded traits, e.g., antibiotic resistance. The range of a plasmid’s promiscuity depends on the adaptations of its transfer, replication, and stability functions to the various hosts. IncU plasmids, with the archetype plasmid RA3, are considered to constitute a reservoir of antibiotic resistance genes in aquatic environments; however, the molecular mechanisms determining their adaptability to a broad range of hosts are rather poorly characterized. Here, we present the transcriptional organization of the stability module and show that the gene transcript dosage effect is an important determinant of the stable maintenance of RA3 in different hosts.

## Abstract (precis)

The efficiently disseminating conjugative or mobilizable BHR plasmids play key roles in the horizontal spread of genetic information between closely related and phylogenetically distant species, which can be harmful from the medical, veterinary, or industrial point of view. Understanding the mechanisms determining the plasmid’s ability to function in diverse hosts is essential to help limit the spread of undesirable plasmid-encoded traits, e.g., antibiotic resistance. The range of a plasmid’s promiscuity depends on the adaptations of its transfer, replication, and stability functions to the various hosts. IncU plasmids, with the archetype plasmid RA3, are considered to constitute a reservoir of antibiotic resistance genes in aquatic environments; however, the molecular mechanisms determining their adaptability to a broad range of hosts are rather poorly characterized. Here, we present the transcriptional organization of the stability module and show that the gene transcript dosage effect is an important determinant of the stable maintenance of RA3 in different hosts.

## INTRODUCTION

Horizontal gene transfer (HGT) is considered the most critical factor in bacterial adaptation and evolution (1–3). Conjugative plasmids play key roles as vehicles in HGT between both closely related and phylogenetically distant species. The broad-host-range (BHR) conjugative plasmids have developed numerous adaptive mechanisms allowing them not only to spread but also to replicate and be stably maintained in a diverse set of hosts (4). The three functional categories of the adaptive mechanisms are responsible, respectively, for conjugation host range, replication host range, and long-term host range, often quite variable in their scope (5). Thus, the conjugation host range refers to the ability to form mating pairs and to disseminate the plasmid genome among various species and is usually the widest host range (6). The replication host range specifies the hosts in which the plasmid can successfully replicate and function as an independent replicon. The long-term host range is generally the narrowest and defines the species in which the plasmid molecules are stably maintained extrachromosomally even in the absence of selective pressure (7).

Studies on the host adaptation mechanisms of BHR plasmids have been focused mainly on their replication systems, the structure of *oriV*s, Rep proteins, and their accessory proteins as well as the interactions between the plasmid and host replication machinery (8–17). Some research has also been done on the factors responsible for the promiscuous conjugative transfer, the role of the ubiquitous type IV secretion systems (T4SS), and the specificity of coupling proteins and primases (18–21). In contrast, relatively little attention has been paid to the adaptation of the stability mechanisms to various hosts in the long-term plasmid persistence (7, 22, 23).

**FIG 1:**
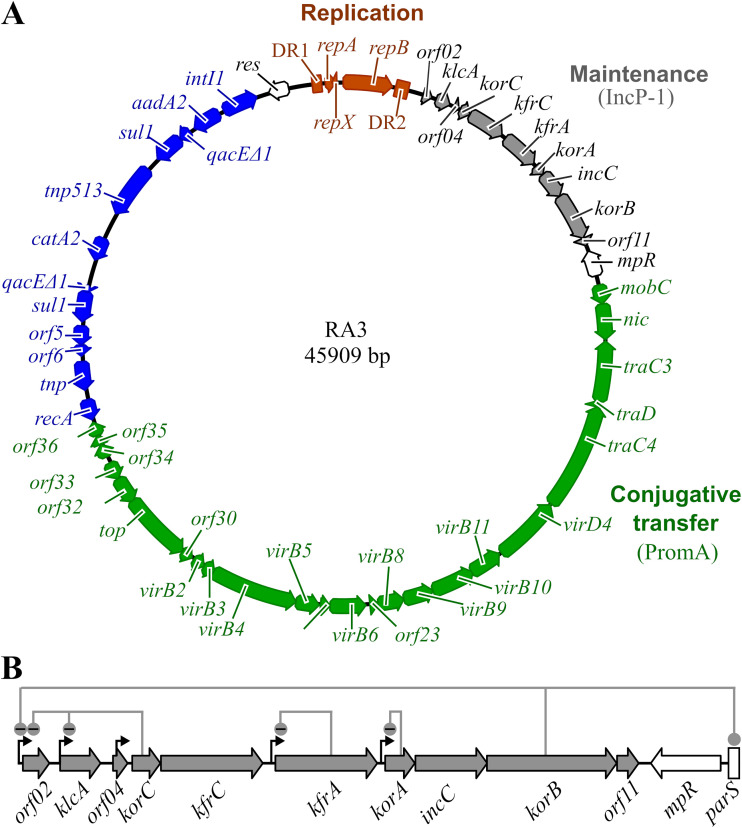
Genomic map of plasmid RA3. (A) Blocks of genes forming distinct functional modules are colored differently. The replication module (brown) is comprised of the *repA-repB* operon surrounded by long direct repeats DR1 and DR2. Genes of the stability module (gray) show homology to IncP-1 plasmids. The conjugative transfer module (green) resembles one found in PromA plasmids. Most accessory genes (blue), so called plasmid “genetic” load, belong to the class I integron (24). Genes *res* (invertase/recombinase) and *mpR* (putative zinc metallopeptidase) of unassigned plasmid functions are labeled in white (24). Arrows indicate the direction of transcription. (B) Close-up of the stability module. Thin black arrows indicate previously identified promoters (24, 28, 29). Regulatory circuits are shown as lines connecting the regulatory genes with their target sequences. KorB binds also to the *parS* centromere-like sequence, a *cis*-acting site of the partition complex.

Our research model is the BHR conjugative plasmid RA3, the archetype of the IncU group, which is capable of replication and stable maintenance in, and transfer between, various representatives of *Alpha*-, *Beta*-, and *Gammaproteobacteria* (24). The RA3 genome, of 49.1 kb (GenBank accession no. DQ401103), has a mosaic modular structure where genes encoding proteins involved in the same process cluster into functional modules, and these modules demonstrate similarity to blocks of genes from various other BHR plasmids (Fig. 1A). The RA3 conjugative module resembles the one from PromA plasmids (25), whereas its stability module encodes proteins homologous to those of IncP-1 plasmids (26, 27).

The stability module of RA3 encompasses the region from nucleotide (nt) 2070 to nt 9830 (Fig. 1B), with 10 open reading frames (ORFs) transcribed in the same direction, i.e., *orf02-klcA-orf04-korC-kfrC-kfrA-korA-incC-korB-orf11*, followed by the centromere-like sequence *parS. In silico* DNA inspection suggested the presence of five transcription initiation signals in this module (24). Cloning of the predicted promoters in the promoter-probe vector and their analysis conducted in Escherichia coli confirmed the functionality of five promoter sequences located upstream of the *orf02*, *klcA*, *korC*, *kfrA*, and *korA* genes, respectively (Fig. 1B). Regulatory studies on individual promoters have revealed that four strong promoters are either controlled by global regulators (*orf02p* and *klcAp*) or efficiently autoregulated (*kfrAp* and *korAp*) (24, 28, 29). Only the weak *korCp* located within putative *orf04* is transcribed constitutively (29).

The best-studied part of the module, the *korA-incC-korB-orf11* operon (28), encodes an active partitioning system, where IncC and KorB belong to the ParA and ParB families of partitioning proteins, respectively, and KorA is a DNA binding protein, an autorepressor of *korAp*. Orf11 fulfills an accessory function in the partition (28). Besides its role in segrosome formation at *parS*, KorB also acts as a global transcriptional regulator controlling stability functions and conjugation by binding to three operator sites (O_B_), i.e., within *orf02p*, the first promoter of the stability module, and two promoters, *mobCp* and *orf023p*, from the conjugative transfer module (29–31). Upstream of the active partition operon are encoded two alpha-helical proteins, KfrA and KfrC, homologs of which were postulated to be involved in the segregation of IncP-1 plasmids (32, 33). KfrA is a DNA binding protein and autoregulates the monocistronic *kfrA* operon (24) whereas KfrC is encoded in the bicistronic *korC-kfrC* operon (Fig. 1B). KorC serves as the main global regulator of plasmid RA3, coordinating the expression of the replication (*orf02p*_rev_), stability (*orf02p* and *klcAp*), and conjugative transfer (*orf033p* and *orf034p*) operons (29). Two monocistronic operons at the beginning of the stability module encode Orf02, a small protein exhibiting similarity to the N-terminal part of DnaA from *Aeromonas* spp., and KlcA, a putative antirestriction protein (34).

The roles of several products of the RA3 stability module have not been fully understood even in E. coli, where the majority of the experiments have been conducted. It also seemed important to decipher how the organization of this unidirectionally transcribed, highly compacted module might influence the expression of the genes in various hosts.

Here, we compared the gene expression patterns of the intact RA3 stability module and its deletion derivatives. Using this approach for the whole RA3 plasmid and also its stability module cloned into a heterologous replicon, we found that this module is organized as a long multicistronic operon with numerous internal promoters and a few terminators/attenuators modulating the expression of downstream genes. The RNA polymerase (RNAP) read-through and an impact of the multiple upstream promoters on downstream gene expression were also detected in hosts other than E. coli, although they varied in different species. The transcriptional studies on RA3 stability module deletion derivatives combined with stability assays revealed the important role of encoded proteins in plasmid maintenance in various hosts.

## RESULTS

### Transcriptional read-through from *orf02p* to *orf11*.

**FIG 2:**
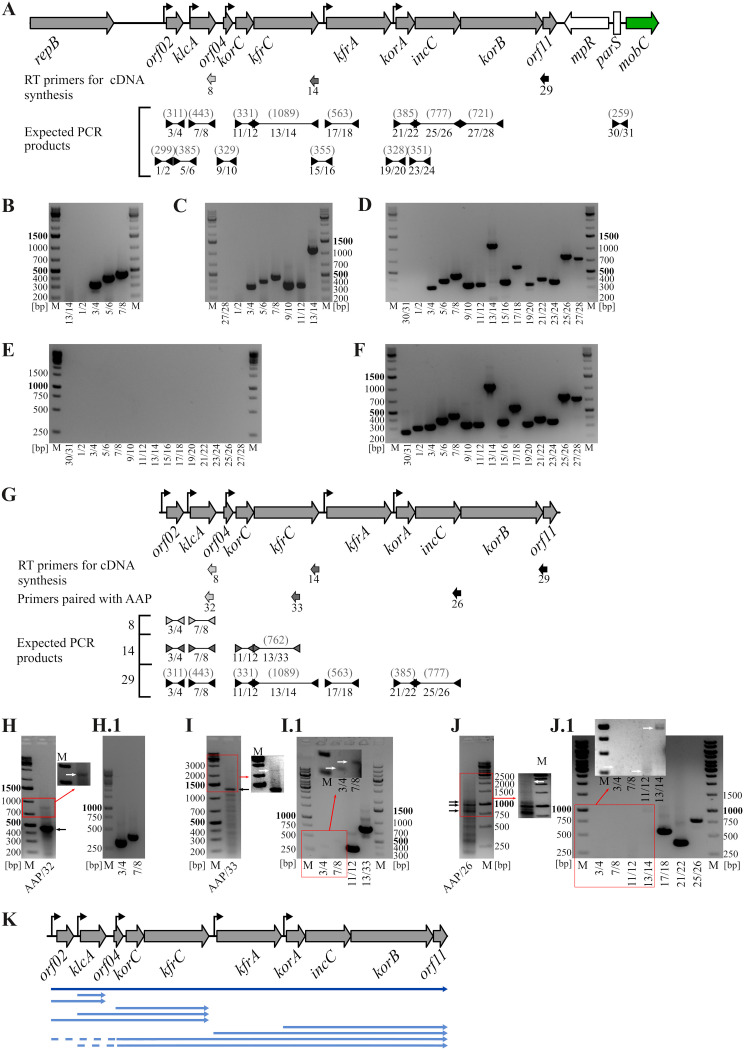
Transcripts of the RA3 stability module. (A to D) Analysis of mRNA by RT-PCR. Total RNA was isolated from E. coli DH5α(RA3) and used for cDNA synthesis. Three primers, 8, 14, and 29, annealing to different parts of the stability module were used in separate reverse transcription (RT) reactions for cDNAs synthesis. The cDNAs were used as templates for PCR with different pairs of primers. PCR-amplified fragments were separated on agarose gels, stained with ethidium bromide, and photographed. (A) Map of the stability module with flanking regions. The positions of RT primers and PCR primers as well as the expected PCR products are shown below. Sizes of PCR products are given in parentheses. (B to D) PCR products obtained with the pairs of primers indicated beneath the lanes and various cDNAs as templates, i.e., synthesized from primer 8 complementary to *klcA* mRNA (B), synthesized from primer 14 complementary to *kfrC* mRNA (C), and synthesized from primer 29 complementary to *orf11* mRNA (D). Next to 1-kb DNA ladder (M) the appropriate control reaction mixture was loaded, i.e., products of PCR with a pair of primers annealing beyond the expected boundaries of the analyzed cDNAs. (E) RNA purity control. PCRs with total RNA as a template instead of cDNAs were run with the indicated pairs of primers. (F) Primer quality control. Products of PCRs with RA3 DNA as a template and the indicated pairs of primers are shown. (G to J) Analysis of mRNAs using 5′RACE. (G) Map of the stability module. The positions of the primers used for cDNA synthesis, the nested PCR primers used in combination with 5′RACE primer AAP (abridged anchor primer), and primers used in the second round of PCRs for three sets of cDNAs are indicated. The sizes of expected PCR products are shown in parentheses. (H to J) Analysis of 5′RACE products obtained in the first set of PCRs with AAP and appropriate nested primers on three cDNAs as templates, i.e., synthesized from primer 8 (H), from primer 14 (I), and from primer 29 (J). Marked sectors of the photographs were manipulated to intensify weak bands (white arrows). The reaction mixtures from panels H to J were used as templates for the second round of PCRs with pairs of specific primers (H.1 to J.1). (K) Schematic summary of the mRNA analysis presented above. Blue arrows depict identified variants of transcripts identified in the above-described experiments. Dashed lines represent deduced parts of the transcripts.

The unidirectional transcription and highly compacted arrangement of the stability module suggested the possibility of reading through from the upstream transcriptional signals into the downstream units. To verify this assumption, total RNA was isolated from the E. coli DH5α(RA3) strain and subjected to reverse transcription followed by PCR (RT-PCR). Three different primers were used in the RT reaction; these were complementary to the 3′ end of the *klcA* gene (primer 8), the 3′ end of the *kfrC* gene (primer 14), and the *orf11* gene (primer 29). The cDNAs obtained in each reaction were then amplified with pairs of primers corresponding to the coding and intergenic parts of the analyzed region (Fig. 2A). PCR products synthesized on a cDNA template started from primer 8 encompassed *orf02* and *klcA* (Fig. 2B), suggesting possibility of RNAP initiating transcription at *orf02p* and reading through the intergenic region *orf02-klcA*. PCR products obtained on cDNAs started from primer 14 indicated the reading through from *orf02p* to *kfrC* (Fig. 2C), whereas the results of PCRs on the cDNA template obtained from primer 29 confirmed that mRNA initiated at *orf02p* might extend through the whole stability region up to *orf11* (Fig. 2D). Notably, no PCR products were obtained in any set of reactions with primers encompassing *orf02p* (primers 1/2), which excludes the transcription from the replication module (Fig. 1 and 2A) into the stability region. For each set of PCRs, an adequate pair of primers downstream of the start site for the given cDNA was used: 13/14 to amplify *kfrC* for the shortest cDNA, 27/28 to amplify *korB*, and 30/31 to amplify the *parS* region for two longer cDNAs (Fig. 2A). For all three cDNAs, the products of such control PCRs were not detected (Fig. 2B, C, and D). Two additional control sets of PCRs were conducted, with RNA as a template (negative control) to verify lack of DNA contamination in RNA samples (Fig. 2E) and with RA3 DNA as a template to demonstrate the efficiency of reaction with all the primers pairs (Fig. 2F).

The RT-PCR results indicated the presence of long transcripts initiated at *orf02p* and continuing toward *orf11*, hence indicating cotranscription of the whole maintenance module. The fact that *orf02*, *klcA*, *korC*, *kfrA*, and *korA* are preceded by functional promoters (24, 28, 29) suggested the possibility of a polarized transcript dosage, with progressively more multiple transcript variants for the genes further downstream in the module. To check if indeed transcripts of various lengths exist for a given ORF, the 5′ rapid amplification of cDNA ends (5′RACE) procedure (35) was applied. Total RNA isolated from E. coli DH5α(RA3) was reverse transcribed with primers 8, 14, and 29 as described above. The cDNAs were 3′ tailed with a stretch of dCTPs and used as templates for PCRs with an abridged anchor primer (AAP) complementary to the dC tail paired with appropriate nested primers 32, 33, and 26, as shown in Fig. 2G. Parts of these RT-PCR mixtures were separated on agarose gels to visualize products corresponding to the cDNAs synthesized on the transcripts that were presumably of various lengths (Fig. 2H, I, and J). The remaining parts were diluted and used as templates for PCRs with pairs of primers specific to the ORFs located upstream of the sequences complementary to primers 32, 33, and 26 (Fig. 2H.1, I.1, and J.1). The best results were obtained for the potential cDNA mixture synthesized with primer 8 complementary to the 3′ end of *klcA*. Two products were visualized in the first set of PCRs with primers AAP and 32, confirming the presence of two transcripts for *klcA* starting at *orf02p* and *klcAp* (Fig. 2H), which was further verified by the subsequent round of PCRs with primers specific to *orf02* and *klcA*, respectively (Fig. 2H.1). When the cDNAs obtained with the use of primer 14, complementary to the 3′ end of the *kfrC* gene, were used as a template, two products were detected after the first round of PCR with AAP and the nested primer 33 (Fig. 2I). The intense band corresponded to the product synthesized on cDNA with the 3′ end determined by the transcription start site (TSS) of *korC*. Another, much weaker band (inset in Fig. 2I) presumably corresponded to cDNA with the 3′ end determined by the TSS of *klcA*. Despite the fact that no product was visible for the cDNA extended up to *orf02p*, the next round of PCRs led to amplification of all four ORFs from *kfrC* up to *orf02*, although with various efficiencies (inset in Fig. 2I.1). The most ambiguous were the results for cDNA(s) obtained with primer 29, which annealed in the region of *orf11*. The PCRs with AAP and the “nested” primer 26 unexpectedly showed the main three products with sizes between 800 and 1,100 bp (Fig. 2J). Whereas a fragment of ca. 1,100 bp might correspond to cDNA ending at the TSS of *korA*, the two smaller products suggested an additional TSS(s) upstream of *incC*. The presence of these putative promoter sequences has been further analyzed (see below). A product of ca. 2,200 bp, corresponding to cDNA ending at the TSS of *kfrA*, was also visible after intensity enhancement (inset in Fig. 2J). The next round of PCRs using the specific pairs of primers for ORFs upstream of *korB* led to the amplification of fragments corresponding to ORFs from *incC* to *orf02* (except for *klcA*), although with the efficiency of the reactions clearly inverse to the distance from the 3′ end of the stability module (Fig. 2J.1). Although the 5′RACE experiments did not directly and clearly show the whole spectrum of transcripts for particular ORFs, they supported the hypothesis of a gradient transcript dosage along the stability module (Fig. 2K). The numerous small PCR products obtained with AAP and “nested primers” likely resulted from nonspecific annealing of the AAP to the C stretches in the GC-rich sequences of the *kfrC*, *kfrA*, and *incC* genes (24).

### Analysis of transcription termination signals.

**FIG 3:**
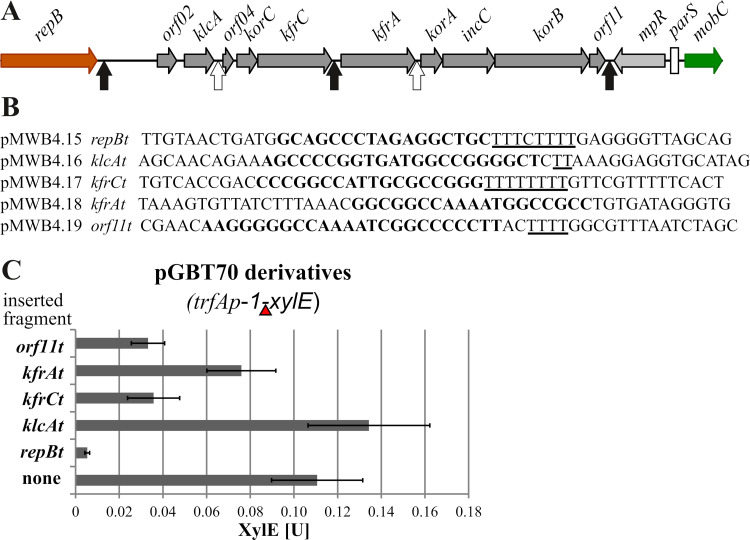
Analysis of putative RA3 transcription terminators in and close to the RA3 stability module. (A) Positions of putative transcription terminators/attenuators in the stability module and flanking regions, with Rho-independent terminators indicated by black arrows and hypothetical transcriptional Rho-dependent terminators/attenuators by white arrows. (B) Putative terminator sequences with GC-rich inverted repeats (bold) and stretches of Ts (underlined) indicated. For *repBt*, a fragment of 157 bp encompassing this sequence was amplified by PCR on RA3 DNA; other sequences were obtained as synthetic double-stranded oligonucleotides. They were cloned into pGBT70 between the *trfAp-1* promoter and the *xylE* cassette (insertion marked by red triangle in panel C). (C) Effect of putative terminators on *xylE* transcription. E. coli DH5α was transformed with pGBT70 (control without additional insert) or its derivatives indicated in panel B. XylE activity was assayed in extracts from exponential-phase cultures of transformants. Experiments were repeated at least three times, and mean values with standard deviations are shown.

The expression of multigenic modules is regulated not only at their promoters but also by transcription terminators. *In silico* inspection of the RA3 replication and stabilization regions uncovered two putative unidirectional Rho-independent transcription terminators (GC-rich palindromic sequence followed by a run of Ts) after the *kfrC* and *orf11* genes in the stabilization module and a third one after the *repB* gene separating the replication and stabilization modules (Fig. 3A and B). Also, the intergenic regions *klcA-orf04* and *kfrA-korA* contain GC-rich palindromic sequences (however, without a long run of Ts) which could pause/modulate the progress of RNAP (Fig. 3A and B). The five relevant intergenic regions were cloned individually into pGBT70 (36) between the strong *trfAp-1*_RK2_ promoter and the *xylE* reporter gene to verify their putative terminator/modulator action. This plasmid had been successfully used to characterize transcription terminator sequences before (37). The plasmid constructs were introduced into E. coli C600K, and the activity of catechol 2,3-dioxygenase, encoded by *xylE*, was assayed. The regions downstream of *repB*, *kfrC*, and *orf11* noticeably hampered the transcription initiated at *trfAp-1*, while the sequence downstream of *klcA* did not (Fig. 3C). The result for the region downstream of the *kfrA* gene was ambiguous, showing a 30% decrease in the XylE activity compared to that for the unmodified pGBT70 plasmid. This decrease may suggest E. coli RNAP pausing at this sequence, but that needs an independent confirmation. Thus, we experimentally demonstrated the presence of two efficient Rho-independent transcription terminators in the RA3 stability module: one in the middle of the module downstream of the *kfrC* gene and the other at the very end of the module, following *orf11*.

### Transcriptional analysis of the synthetic RA3 stability module and its mutated variants: effect of mutations on plasmid stability in various hosts.

To assess the roles of the individual promoters in the expression of downstream genes/operons in the RA3 stability module, a set of deletion variants, deprived of one or a combination of several promoters and/or particular genes, was constructed (see the supplemental material). The obtained library of the module variants, cloned into an unstable BHR plasmid, allowed study of the roles of genes of interest in the maintenance of the plasmid in various hosts.

**FIG 4:**
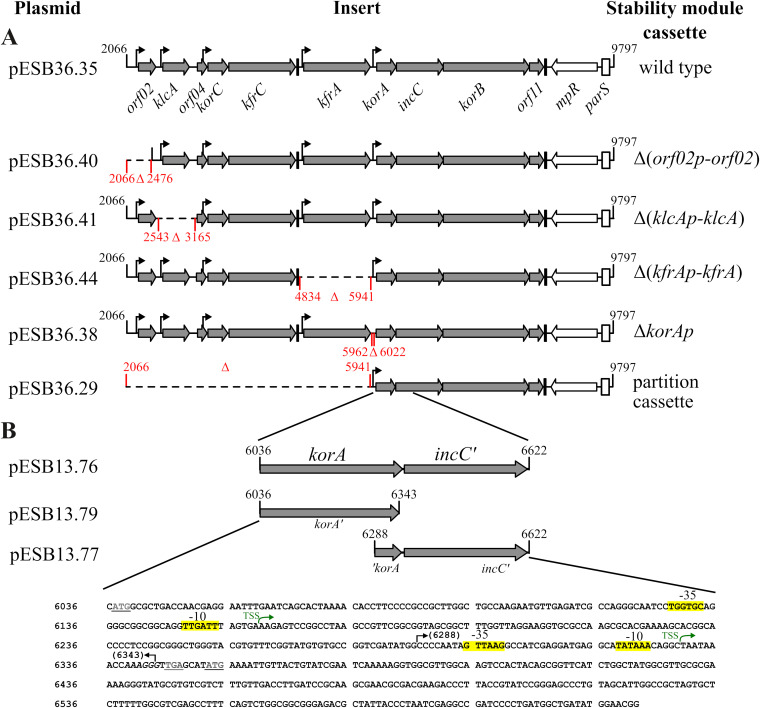
Deletion variants of the RA3 stability module used for studies on gene expression and plasmid maintenance in diverse hosts. (A) Schematic presentation of the stability module variants cloned into the low-copy-number pESB36. Thin black arrows indicate known promoter sequences, and black bars mark positions of Rho-independent transcriptional terminators. The extent of deletions is shown in red. (B) Identification of previously unanticipated promoters within the *incC* gene. The indicated regions were cloned into the promoter-probe vector pPT01 upstream of the *xylE* cassette and introduced into E. coli DH5α. Activity of the putative promoters was analyzed by XylE assay (see the text). The nucleotide sequence of the fragment cloned into pESB13.76 is presented. The black arrows delineate boundaries of the smaller fragments. The stop codon for *korA* and the start codon of *incC* are underlined. Green arrows depict two newly identified TSSs for *incC* in the *korA* coding sequence. Putative promoter motifs are highlighted in yellow.

For these studies, the wild-type (WT) module and its Δ(*orf02p-orf02*), Δ(*klcAp-klcA*), Δ(*kfrAp-kfrA*), Δ*korAp*, and Δ(*orf02p-kfrA*) (the partition operon on its own) variants were chosen (Fig. 4A). The rationale behind the deleting of the promoter regions with the adjacent ORFs in the case of the monocistronic and strongly regulated *klcA* and *kfrA* operons was to avoid altered expression of these presumably important ORFs from upstream promoters. As a precaution, no genetic manipulations were undertaken in the *korC* operon, since KorC is a potent transcriptional regulator of *orf02p* and *klcAp* (29). The constructed modules were cloned into a single-copy, extremely unstable pESB36 vector, a derivative of pABB32 (38). pESB36 is based on the RK2 minireplicon (pRK415), is mobilizable by the RK2 conjugative system owing to the presence of *oriT*_RK2_, and carries a convenient system for detection of the plasmid presence in colonies (*repA*p_RA3_-*lacZ* transcriptional fusion). pESB36, pESB36.35 (WT stability module inserted), and its deletion derivatives (Fig. 4A) were introduced into two representative *Gammaproteobacteria* species (E. coli and Pseudomonas putida), two *Alphaproteobacteria* species (Agrobacterium tumefaciens and Paracoccus aminovorans), and one *Betaproteobacteria* species (Cupriavidus necator). Transformants/transconjugants were analyzed with respect to stability module gene expression and stable maintenance of the pESB36.35 derivatives during growth without selection. The plasmid segregation assay demonstrated that the pESB36 loss rate (LR) was variable in the hosts tested and varied from 2% per generation in P. putida to 18% per generation in E. coli and to more than 25% per generation in P. aminovorans (Table 1). Such differences in the vector stability could be due to the variations in the plasmid copy number (PCN) caused by differential gene expression of the miniRK2 replicon in the analyzed species. Estimation of the pESB36 copy number per chromosome in cells from stationary-phase cultures grown under selection confirmed this conjecture. The PCN for two hosts, P. aminovorans and E. coli, was only 0.12 to 0.15, whereas that for P. putida was 2.9 (Table 2).

**TABLE 1:** Loss rate per generation and stability index of analyzed derivatives of pESB36 in various hosts^*a*^

Plasmid	LR and SI in host:									
	Escherichia coli		Paracoccus aminovorans		Agrobacterium tumefaciens		Cupriavidus necator		Pseudomonas putida	
	LR (%)	SI	LR (%)	SI	LR (%)	SI	LR (%)	SI	LR (%)	SI
pESB36	18.0	X	>25	X	2.7	X	4.9	X	2.0	X
pESB36.35 (WT)	3.5	5.1	0.4	NC	0.5	5.4	1.1	4.9	0.5	4.0
pESB36.40 (Δ*orf02p-orf02*)	14.5	1.2	0.05	NC	1.8	1.5	4.6	1.1	2.2	0.9
pESB36.41 (Δ*klcAp-klcA*)	15.0	1.2	0.3	NC	1.0	2.7	5.8	0.8	2.0	1.0
pESB36.44 (Δ*kfrAp-kfrA*)	11.4	1.6	0.5	NC	1.2	2.3	5.0	1.0	2.4	0.8
pESB36.38 (Δ*korAp*)	4.6	3.9	ND	ND	ND	ND	ND	ND	2.7	0.7

a X, irrelevant; NC, not calculable; ND, not determined.

**TABLE 2:** Estimated plasmid copy numbers in various hosts

Plasmid	Mean (SD) copy no. in host:				
	Escherichia coli	Paracoccus aminovorans	Agrobacterium tumefaciens	Cupriavidus necator	Pseudomonas putida
pESB36	0.15 (0.02)	0.12 (0.03)	1.03 (0.12)	2.62 (0.54)	2.9 (0.68)
pESB36.35	1.31 (0.09)	1.08 (0.17)	3.08 (0.70)	3.34 (0.67)	3.3 (0.69)

The presence of the RA3 stability module significantly increased the persistence of the plasmid (pESB36.35) in all the species tested, again to different extents. The plasmid was very stably maintained in P. aminovorans, P. putida, and A. tumefaciens, with a loss rate per generation of 0.4 to 0.5% (Table 1), and slightly less so in C. necator (LR, 1.1%). The highest plasmid loss rate, 3.5%, was found for E. coli. However, the ratio of the loss rates for the empty vector and pESB36.35 in a given strain, the so-called stability index (SI), was quite similar in all the hosts, with the exception of P. aminovorans, where it exceeded 60 (Table 1).

**(i) Expression of the stability module and its deletion variants in E. coli.** The E. coli EC1250 (Δ*lac*) strain was transformed with pESB36, pESB36.35, and its derivatives, transformants were grown on rich medium with selection, and total RNA was isolated. cDNA was synthesized on the RNA with the use of a mix of random hexamer primers, and then quantitative real-time PCR (qPCR) was performed with pairs of primers specific for each ORF of the stability module, with the exception of the short *orf04* overlapping *korCp*. The results were normalized to the amount of mRNA for the chromosomal marker *cysG* (39).

**FIG 5:**
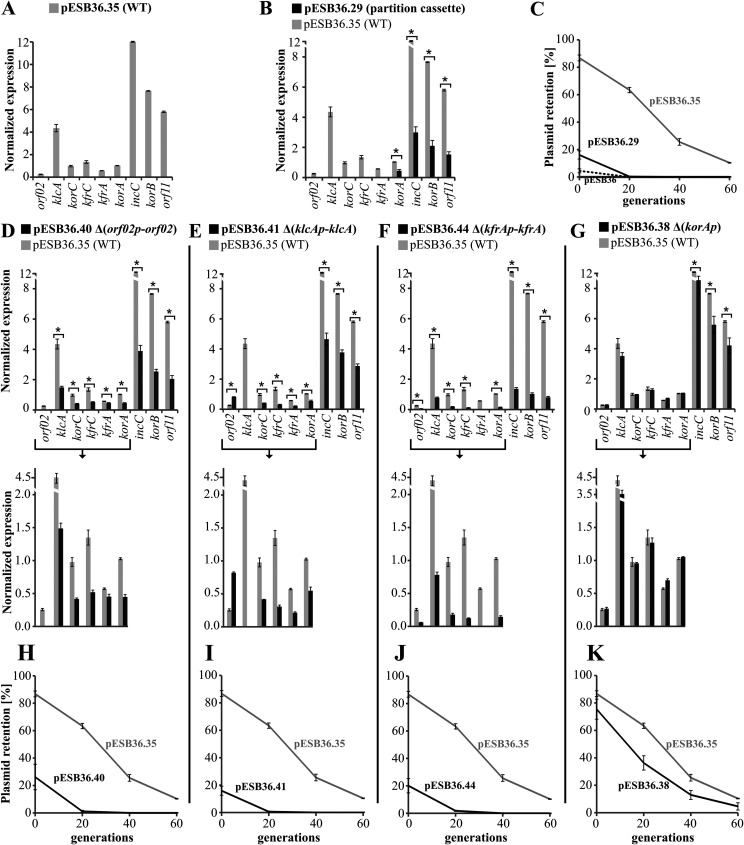
Transcription profiles of RA3 stability module deletion variants in E. coli. E. coli strain EC1250 was transformed with the pESB36 vector carrying RA3 stability module variants as indicated. Total RNA was isolated from three exponentially growing cultures of transformants in the presence of selection (biological replicates) and used as the template for cDNA synthesis with random hexameric primers. Three technical replicates of qPCRs with pairs of primers corresponding to individual ORFs were run for each cDNA lot. Target gene expression was normalized to the expression of a chromosomal reference gene, *cysG*. Mean values with standard deviations for at least three assays are shown. (A) Normalized expression of the ORFs in the WT stability module [E. coli EC1250(pESB36.35)]. (B and D to F) Normalized expression of ORFs in deletion variants (dark gray bars) was compared to that in the WT stability module (light gray bars). The bracketed parts of the diagrams were scaled up for clarity. E. coli EC1250 carrying the following plasmids was studied: pESB36.29 (Δ*orf02p-kfrA*, partition operon), pESB36.40 (Δ*orf02p-orf02*), pESB36.41 (Δ*klcAp-klcA*), pESB36.44 (Δ*kfrAp-kfrA*), and pESB36.38 (Δ*korAp*). *, *P* < 0.05 in two-sided Student *t* test, assuming equal variance. (C and H to K) Plasmid segregation assays. Transformants of E. coli strain EC1250 were grown without selection for up to 60 generations. Every 20 generations, cultures were spread on L agar with X-Gal. Plasmid retention was expressed as the relative number of blue colonies.

The expression of blocks of genes in the WT stability module varied significantly, confirming the functionality of the five previously identified promoters in the context of the whole module (Fig. 5A). The lowest levels of transcripts were found for *orf02*, the first ORF in the module, and for *kfrA*, both presumably encoded by the tightly regulated monocistronic operons (24). In turn, *klcA* expression was 10-fold higher than that of *orf02*, despite the expected strong repression of *klcAp* by KorC (29). The *korC*, *kfrC*, and *korA* genes showed an intermediate level of expression, and unexpectedly, the highest levels of transcripts were found for *incC*, *korB*, and *orf11*.The organization of the *korA-incC-korB-orf11* region suggested a single operon structure (with closely packed or overlapped ORFs) whose expression was dependent on the strong but autorepressed *korA* promoter (24, 28). The observation here of decoupling of *korA* expression and three downstream genes raised the possibility of the existence of an internal promoter(s) in this operon. The presence of the promoters driving a *korAp*-independent expression of *incC-korB-orf11* (at least in E. coli) was verified by cloning of a 586-bp DNA fragment (Fig. 4B) encompassing the *korA* coding sequence and the 5′ end of *incC* into the promoter-probe vector pPT01 (40) upstream of the *xylE* cassette (pESB13.76). Determination of the XylE activity in the extracts from exponentially growing E. coli C600K(pESB13.76) cells revealed a weak transcriptional activity of the cloned fragment [7.2 mU, versus 0.5 mU in control E. coli C600K(pPT01) cells]. A similar XylE activity (6.5 mU) was detected in extracts from E. coli C600K(pESB13.77) and C600K(pESB13.79) when the shortened fragments were cloned upstream of *xylE*, pointing out the localization of the *incC* promoter(s) in the 3′ end of *korA* (Fig. 4B). Importantly, the 5′RACE experiments also suggested the possibility of additional TSSs within *korA* (Fig. 2J). The products detected in the 5′RACE experiments were isolated and sequenced. The 5′ ends of these fragments localized two putative TSSs in *korA*, one corresponding to the 3′ end of *korA* and another 169 bp upstream, as marked in Fig. 4B together with corresponding promoter motifs. Importantly, these weak constitutive internal transcriptional signals seem to significantly increase the amounts of mRNAs for partitioning genes in comparison to *korA* mRNA (Fig. 5A). Such accumulation of shorter transcripts may result from the higher stability of these mRNAs than those initiated at *korAp*, as differential mRNA decay has been observed in other prokaryotic multicistronic operons (41, 42).

The transcription profiling of the stability module variants lacking single or several promoters confirmed the participation of RNAP read-through in the establishing of this region’s expression pattern (Fig. 5B to F). It was most clearly seen when the expression of the *korA-incC-korB-orf11* genes was compared between the intact WT stability module and the partition operon on its own (Δ*orf02p-kfrA* variant, pESB36.29) (Fig. 5B). The transcripts for the partition operon genes were almost four times less abundant in the construct lacking all the DNA sequence upstream of *korAp* than in the WT stability module. Similarly, deletion of *orf02p-orf02* at least halved the expression levels of the majority of downstream genes compared to the WT module (Fig. 5D). Deletion of the *klcAp-klcA* region decreased the expression of downstream genes 2- to 3-fold but, in parallel, increased the expression of *orf02* 3-fold (Fig. 5E). The apparent derepression of *orf02p* might be due to the lower level of expression of *korC* and *korB*, encoding *orf02p* repressors (29). Another plausible explanation of the observed “induction” might be a relief from a negative interference between the *orf02p* and *klcAp* transcriptional and regulatory signals, since these two promoter regions arose by duplication (24). The unexpected lack of transmission of the enhanced *orf02p* activity to the genes downstream of *orf02* could be a result of the genetic manipulations leading to the deletion of the *klcAp-klcA* region.

In the absence of *korAp*, no change in expression of *korA* was detected and there was only a slight decrease in the expression of the partitioning genes (Fig. 5G), confirming significant participation of RNAP read-through from the upstream promoters into *korA* and a minor role of *korAp* in driving expression of downstream loci. The most unexpected result was the strong effect of the *kfrAp-kfrA* deletion on the expression of the whole stability module (Fig. 5F). Genes downstream of *kfrA* were even expressed 12-fold less abundantly despite the low level of transcriptional activity of *kfrAp* in the intact module. Intriguingly, the upstream genes also were strongly downregulated. Further studies should elucidate the consequences imposed on plasmid functions by the lack of *kfrAp-kfrA*.

E. coli EC1250 (Δ*lac*) transformants carrying pESB36.35 variants were also tested for plasmid retention over 60 generations of growth in rich medium without selection. Analysis of plasmid stability showed that all deletion variants except pESB36.38 Δ*korAp* were unstable in E. coli (Fig. 5C and H to K). Since only the Δ*korAp* derivative demonstrated hardly any effect on expression of the partition operon, it may be concluded that any manipulation leading to the decreased transcription of *incC-korB-orf11* led to an increase in the rate of loss of these plasmids (Fig. 5 and Table 1).

**FIG 6:**
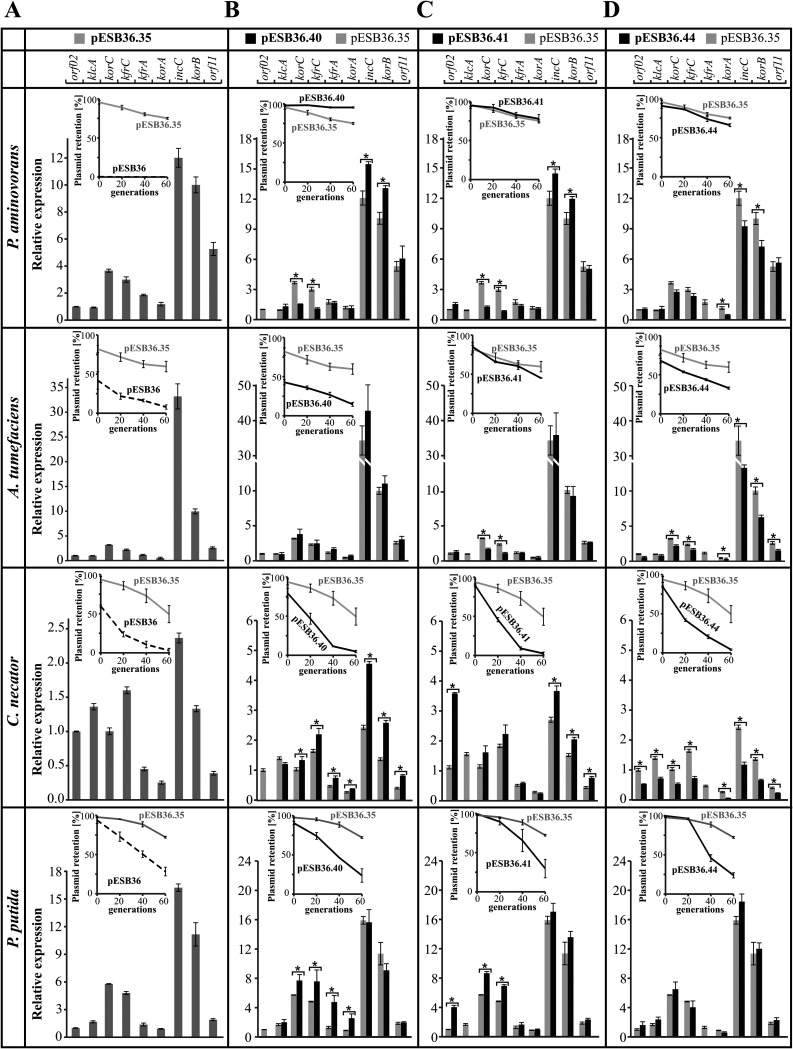
Transcriptional profiles of RA3 stability module variants in Paracoccus aminovorans, Agrobacterium tumefaciens, Cupriavidus necator, and Pseudomonas putida hosts. RT-qPCR analysis was conducted on RNA samples isolated from exponentially growing cultures of transconjugants of appropriate hosts. Expression of stability module ORFs was normalized to the *rpoD* reference gene in P. aminovorans, A. tumefaciens, and P. putida and to *gyrB* in C. necator. Mean values with standard deviations for at least three assays are shown. (A) Relative expression of the ORFs in the WT stability module (pESB36.35) in various hosts. For clarity of presentation, the normalized results (target gene versus reference gene for each strain) are shown relative to the expression of *orf02* in each strain. Insets demonstrate pESB36.35 plasmid retention in comparison to vector pESB36 in the analyzed host. (B to D) Relative gene expressions in the WT stability module (pESB36.35, light gray bars) and tested deletion variants (dark gray bars) in each of the hosts. For clarity of presentation, the normalized results are shown relative to the expression of the *orf02* from the WT stability module (pESB36.35) in each strain. Insets demonstrate retention of each plasmid variant in the analyzed host in comparison to vector pESB36.35 carrying the intact stability module (WT). *, *P* < 0.05 in two-sided Student *t* test, assuming equal variance.

**(ii) Expression of the stability module in hosts other than E. coli.** The RA3 plasmid has a very wide range of hosts belonging to *Alpha*-, *Beta*-, and *Gammaproteobacteria* (24), and hence it was important to establish whether the transcriptional signals characterized in E. coli function in a similar way in the other hosts and whether the RNA polymerases of other bacteria were also capable of reading through the termination/attenuation signals in the stability module. The mRNA levels of the RA3 genes of interest were measured with respect to those of the recommended reference gene for each transconjugant, and the results are presented in Fig. 6, normalized to *orf02* taken as 1. The analysis of the WT stability module expression (pESB36.35) by RT-qPCR demonstrated that in all transconjugants the levels of *incC* and *korB* transcription were the highest (or among the highest, as in C. necator), similarly to the case in E. coli, but the expression of the genes preceding the partition operon varied significantly depending on the host (Fig. 6A). The expression of *orf02* and *klcA* seemed to be correlated and severalfold lower than that of *korC* and *kfrC* in P. aminovorans, A. tumefaciens, and P. putida, whereas in C. necator the first four genes were expressed at comparable levels. Expression of *kfrA* was like the expression of *orf02* and *klcA* in A. tumefaciens and P. putida, lower in C. necator, and slightly higher in P. aminovorans. These results indicated substantial modulations of the abilities of the host transcriptional machinery to recognize different initiation signals (e.g., more efficient expression from *korCp* in all species other than E. coli) and presumably to respond to regulators such as KorC (*orf02p* and *klcAp*) or KfrA (*kfrAp*).

To check whether read-through takes place in hosts other than E. coli, the expression of genes in the Δ(*orf02p-orf02*), Δ(*klcAp-klcA*), and Δ(*kfrAp-kfrA*) variants of the stability module was analyzed by RT-qPCR in the transconjugants of four hosts carrying pESB36.40 (Fig. 6B), pESB36.41 (Fig. 6C), and pESB36.44 (Fig. 6D), respectively. Plasmid retention in transconjugants was also monitored for 60 generations of growth without selection, and the results of the stability assays are shown in insets in Fig. 6. Additionally, the calculated plasmid loss rates and stability indexes (if feasible to calculate) are included in Table 1.

Expression of the truncated stability modules varied between the strains. Notably, the deletion of *orf02p-orf02* did not have as strong a negative effect on the level of transcripts of the downstream genes as in E. coli (Fig. 5D and 6B). A decrease in transcription, but only of *korC* and *kfrC*, was observed in P. aminovorans (with a simultaneous increase in the transcription of the partition operon), and no change in expression of the stability module was observed in A. tumefaciens (Fig. 6B, two upper panels). In contrast, in C. necator the *orf02p-orf02* deletion had a positive effect on the expression of almost all remaining genes (with the highest effect on the partition operon), whereas an increase in *korC*, *kfrC*, *kfrA*, and *korA* expression (but not that of *incC-korB-orf11*) was detected in P. putida (Fig. 6B, two bottom panels). This may suggest that RNAP read-through from *orf02p* encompasses only *klcAp* and *korCp* in P. aminovorans, plays no role in A. tumefaciens, and negatively interferes with the expression of downstream operons in C. necator and P. putida. Expression of the partition operon seems to be uncoupled to some extent from other parts of the module in these four species. Notably, deletion of the *orf02p-orf02* fragment had a large impact on pESB36.40 stability in the analyzed hosts (Fig. 6B and Table 1). Similar to the case for E. coli (Fig. 5D), the construct was unstable in A. tumefaciens, C. necator, and P. putida (loss rate close to that of pESB36 vector). In E. coli the pESB36.40 loss might have resulted from the lower expression of the partition operon (or of other genes in the module) (Fig. 5D and H). In species in which the remaining genes in the stability module were expressed at the same level as in the wild-type fragment (A. tumefaciens) or even higher (C. necator and P. putida), the instability was likely caused by lack of the Orf02 product itself. The reverse effect of the *orf02p-orf02* deletion on plasmid stability was observed in P. aminovorans, in which the pESB36.40 loss rate dropped 8-fold. This coincides with the increase of the partition operon expression but also with the decrease in the level of *korC* and *kfrC* transcripts. Further studies should define the role of Orf02 in regulation of gene expression and stability of the plasmid in these hosts.

The *klcAp-klcA* deletion led to a severalfold increase of the transcription from *orf02p* in E. coli (Fig. 5E). A similar effect was observed in C. necator and P. putida (Fig. 6C); however, in these hosts, in contrast to the case for E. coli, it was conveyed into higher expression of *korC-kfrC* operon, as expected for the RNAP reading through from *orf02p* into downstream genes. In two other species, P. aminovorans and A. tumefaciens, the lack of the *klcAp-klcA* DNA fragment lowered the transcription of the *korC-kfrC* operon, indicating significant participation of *klcAp* in the expression of this part of the stability module. Remarkably, the deletion of *klcAp-klcA* fragment either had no impact on the expression of the partition operon (A. tumefaciens and P. putida) or led to an increase of the expression of this operon (P. aminovorans and C. necator). In these four strains, the effect of *klcAp-klcA* deletion on the expression of the adjacent operon, *korC-kfrC*, varied from the effect on expression of the partition operon, confirming the uncoupling of two parts of the stability module (Fig. 6C).

The maintenance of the construct deprived of *klcAp-klcA* (pESB36.41) varied from being stable in P. aminovorans and A. tumefaciens to being unstable in C. necator and P. putida (insets in Fig. 6C and Table 1) and E. coli (Fig. 5I). Instability of the construct in E. coli might be explained by lowered expression of the partition operon; however, in C. necator the partition operon transcription was elevated, and in P. putida it was unchanged, in comparison with the WT module. Hence, either increased production of Orf02 or lack of KlcA may account for the plasmid instability in these hosts. Further studies are needed to discriminate between these options. It is worthwhile to notice that deletions of either *orf02p-orf02* or *klcAp-klcA* had an identical impact on the stability module expression in P. aminovorans (Fig. 6B and C, upper panels); however, only the first deletion increased plasmid stability in this host. This favors the hypothesis of Orf02 playing the negative role in plasmid maintenance in this species.

Deletion of *kfrAp-kfrA* in E. coli had a significant influence on expression of the upstream and downstream genes in the module (Fig. 5F). A similar effect of Δ(*kfrAp-kfrA*) was detected in C. necator and A. tumefaciens (Fig. 6D). In P. aminovorans only expression of the partition operon was significantly lowered, but it did not affect the stability of pESB36.44 in this host. Modest instability of the construct was observed in A. tumefaciens, whereas the highest plasmid LR was observed in C. necator and P. putida (Fig. 6D and Table 1). The decrease in pESB36.44 stability in A. tumefaciens, C. necator, and E. coli (Fig. 5J) may result from the lower expression of the partition operon or other genes; however, in the case of P. putida, the lack of KfrA itself seems to be deleterious for the plasmid maintenance (Fig. 6D, insets, and Table 1).

## DISCUSSION

Plasmids usually provide advantageous traits to their hosts but also impose a fitness cost on the cells. In the absence of selection for the plasmid-borne beneficial traits, the cells that have lost the plasmid easily outcompete the plasmid-bearing rivals (43), which should lead to plasmid extinction. However, laboratory and environmental studies have shown long-term plasmid persistence under nonselective conditions, a phenomenon dubbed the “plasmid paradox” (44). To ameliorate the fitness cost, the plasmid and host genomes undergo compensatory coevolution, which has been of great research interest in recent years (17, 43, 45–48). To increase the probability of the compensatory evolution events taking place, plasmids have to ensure lowering the rate of their loss (49, 50).

To this end, plasmids have evolved efficient copy number control mechanisms and tight regulation of gene expression to diminish the metabolic burden imposed on the host as well as evolved/acquired specific stability mechanisms to support their maintenance in the population, which is especially important for low-copy-number replicons. Besides the prevalent and well-known multimer resolution systems (51), active partition mechanisms (52), and postsegregational killing (toxin-antitoxin) systems (53, 54), additional factors may play significant roles during the establishment of broad-host-range plasmids in phylogenetically diverse hosts (7, 45, 55).

Above all, the promiscuous plasmids have to secure an adequate production of vital plasmid proteins regardless of the highly diversified efficiency of the transcriptional and translational machinery in these hosts (56). The question of how the broad-host-range plasmids cope with various host factors to ensure adequate levels of plasmid gene expression is far from being fully understood.

In general, gene expression depends on the RNAP recognition of promoters (57), the level of transcriptional regulators, transcriptional organization, global and local DNA topology controlled by host- or plasmid-encoded nucleoid-associated proteins (NAPs) (58, 59), and the position of the gene in the genome with respect to the origin of replication (60, 61). The initial concept of the bacterial operon defined it as a transcriptional unit (TU) that provided simultaneous expression of genes within the operon through production of a single polycistronic mRNA initiated at the promoter upstream of the first gene (62). Control of this unique promoter allowed a coordinated regulation of all genes. However, recent studies have revealed that bacterial transcriptomes are far more complex than previously thought (63–65). The architecture of bacterial operons may involve multiple internal differently regulated promoters and various terminators generating multiple TUs and leading to differential gene expression within these operons (66). It has been shown that various blocks of genes in an operon are often alternatively transcribed under diverse conditions; e.g., the E. coli polycistronic flagella operon *fliF* to *fliR* of thirteen genes may be split in up to seven various suboperons after heat shock or phosphorus starvation (67).

Genome-wide transcript analysis not only demonstrated variability of transcripts corresponding to the operon-clustered genes but also stressed the important regulatory roles that the stability of the mRNAs and their translation efficiency play in gene expression (42, 66, 68–71). First, it was assumed that the prokaryotic transcripts are processed only by 3′-to-5′ exonucleases, leading to an abundance of transcripts for the 5′ part of the multicistronic operons (polarity expression). Discovery of the endoribonucleases and their role in mRNA decay in combination with regulatory functions of RNA binding proteins and noncoding RNAs (ncRNAs) revealed a complex regulatory network responsible for differential transcript abundance even in the operons (72–76).

Similarly to the organization of their hosts’ genomes, the mosaic plasmids cluster the genes engaged in the same processes into functional modules, i.e., the replication, conjugation, and stability modules. This evolutionary trend has been usually explained by gene products forming complexes, e.g., replisome, relaxosome, or transferosome, and temporal/spatial requirements for the synthesis of such complexes (77). Even if various stability functions of the plasmids are encoded in several operons, they are often located next to each other, seemingly representing separate TUs integrated only by common regulators, such as in RK2 (GenBank accession no. BN000925) of IncP-1 (78), R388 (GenBank accession no. BR000038) of IncW (79), or R46 (GenBank accession no. AY046276) of IncN (80).

Our studies on plasmid RA3, the archetype of the IncU group, have revealed that this conjugative, low-copy-number, broad-host-range plasmid has a mosaic modular structure with functional modules intertwined by the global regulatory network (24, 28–31, 81). The present project focused on the expression of the RA3 stability module, comprising a unidirectionally transcribed set of ten ORFs split apparently into five differently regulated operons (24). In the course of these studies, additional, previously unanticipated TSSs as well as transcription terminator and attenuator signals were found in the RA3 stability module.

The active partition operon *korA-incC-korB-orf11* has previously been identified as the main part of the stability module (24, 28), but the roles of the remaining six ORFs have not been known. Before attempting to analyze them, it seemed important to establish the transcriptional organization of the region and elucidate if and how the individual regions of the module influence the expression of other genes. The possibility of RNAP read-through and its putative effect on the relative abundance of individual transcripts along the module (gene transcript dosage) were investigated.

Thus, it was shown here that the whole RA3 stability module was built as a polycistronic operon, *orf02* to *orf11*, but transcribed into alternative mRNAs due to at least five internal promoters and various terminators. Such a transcriptional organization may have evolved to ensure the relative expression levels of the individual genes that would meet the requirements of various hosts. The transcription analysis of the stability module in representative strains of *Alpha*-, *Beta*-, and *Gammaproteobacteria* in which RA3 replicated and seemed to be stably maintained (24) indicated differential expression patterns.

The transcriptional profiles of the RA3 stability module in E. coli and the four other hosts, P. putida, P. aminovorans, A. tumefaciens, and C. necator, showed similar abundances of transcripts for the partition genes (Fig. 5A and 6A) but a substantial variability for the upstream region of the module. The relatively high expression from *korCp* in P. putida, P. aminovorans, and A. tumefaciens suggests more efficient RNAP recognition of this transcription initiation signal, leading to a stronger repression of the first two promoters by KorC than in E. coli (Fig. 5D and 6B). In turn, in C. necator the expression of the first three operons was at a similar level, indicating yet another specificity of its transcriptional machinery.

Transcriptional analysis of pESB36.35 deletion derivatives in E. coli clearly demonstrated the impact of the upstream transcriptional signals on the expression of the downstream genes due to RNAP read-through. Some observations indicated that multiple transcripts originating in the stability module may be more prone to nucleolytic degradation than others, as was observed, e.g., for the active partition operon. The much higher level of *incC* mRNAs than of *korA* mRNAs not only seems to result from the presence of weak internal promoters within *korA* but indicates the distinct stability of transcripts starting at *korAp* versus *incCp*.

The transcriptional studies conducted in different hosts demonstrated the species-specific dependence of expression of particular cistrons on RNAP read-through, TSSs, and intrinsic and Rho-dependent terminators and also suggested differential mRNA decay. In various combinations of host and plasmid deletion variant, a high plasmid loss rate was observed (Table 1), but further studies are needed to define whether the plasmid instability is due to transcriptional dysfunction or lack or abundance of specific products.

Analysis of the stable maintenance of pESB36.35 deletion derivatives in various hosts combined with the transcriptional analysis by qRT-PCR allowed us to determine the important, though converse, roles of the Orf02 product in A. tumefaciens, C. necator, P. putida, and P. aminovorans. At this point, no conclusion about Orf02 function in E. coli may be drawn, since deletion of *orf02p-orf02* diminished expression of all downstream genes and the effect on segregation might have been caused by lower expression of, e.g., the partition operon. As mentioned above, the destabilizing effect of KlcA deficiency in E. coli, C. necator, and P. putida is far more complicated to interpret due to the simultaneous increase in *orf02* expression. Finally, KfrA is definitely important for plasmid establishment/maintenance in P. putida, E. coli, A. tumefaciens, and C. necator but not in P. aminovorans, and its role is under investigation.

Studies on functions of particular plasmid-encoded proteins in plasmid biology are usually carried out on deletion/substitution mutants. However, as shown here, such genetic modifications may introduce changes in the transcriptional organization (initiation and termination signals) and regulation of gene/operon expression locally but may also affect gene expression in other regions of the plasmid. To avoid erroneous conclusions, the analysis of plasmid functions should be complemented by a thorough transcriptional analysis. As demonstrated here, in the case of broad-host-range plasmids, such analysis should be conducted in diverse hosts.

## MATERIALS AND METHODS

### Bacterial strains and growth conditions.

The Escherichia coli strains used were C600K (*thr-1 leu-6 thi-1 lacY1 supE44 ton21 galK*) (82), DH5α [F^−^ (ϕ80d*lacZ*Δ*M15*) *recA1 endA1 gyrA96 thi-1 hsdR17*(r_K_^−^ m_K_^+^) *supE44 relA1 deoR* Δ(*lacZYA-argF*)*U169*] (83), EC1250 [F^−^ *araD139* Δ(*lacZYA-argF*)*U169 rpsL150 relA1 deoC1 ptsF25 rbsR flbB5301 trp-1*] (84), and S17-1 (*recA pro hsdR* RP4-2-Tc::Mu-Km::Tn*7*) (85). Agrobacterium tumefaciens LBA1010R Rif^r^ and Paracoccus aminovorans JCM7685 Rif^r^ were kindly provided by D. Bartosik (University of Warsaw, Poland). Pseudomonas putida KT2442 Rif^r^ was kindly provided by C. M. Thomas (University of Birmingham, United Kingdom). Cupriavidus necator (previously Ralstonia eutropha) 7MP228r Rif^r^ was kindly provided by K. Smalla (Research Institute for Cultivated Plants, Germany).

Bacteria were grown in L broth (86) at 37°C (E. coli) or 28°C (A. tumefaciens, C. necator, P. aminovorans, and P. putida). L broth or L agar (L broth with 1.5% [wt/vol] agar) were supplemented with kanamycin (Km) (50 μg ml^−1^ for E. coli and 20 μg ml^−1^ for P. aminovorans and P. putida), chloramphenicol (Cm) (10 μg ml^−1^ for E. coli, 50 μg ml^−1^ for A. tumefaciens, and 150 μg ml^−1^ for C. necator), and rifampin (Rif) (100 μg ml^−1^). The L agar used for blue/white screening contained 40 μg ml^−1^ X-Gal (5-bromo-4-chloro-3-indolyl-β-d-galactopyranoside).

### Plasmid DNA isolation and analysis and DNA amplification and manipulation.

Plasmids used and constructed in this study are listed in Table 3 (those referred to in the main text) and in Table S1 in the supplemental material (intermediate constructs). Plasmid DNA manipulations were carried out by standard procedures (87) and in accordance with the manufacturers’ instructions. Standard PCRs (88) were performed with appropriate pairs of primers listed in Table 4 and the RA3 DNA template. Complementary oligonucleotides corresponding to the putative transcriptional terminator sequences or introducing restriction sites were annealed by heating to 95°C, slowly cooled, and cloned into appropriately digested plasmids. All new plasmid inserts were verified using dye terminator sequencing at the Laboratory of DNA Sequencing and Oligonucleotide Synthesis, Institute of Biochemistry and Biophysics, Polish Academy of Science.

**TABLE 3:** Plasmids used and constructed in this study that are mentioned in the text

Plasmid	Relevant features or description (reference)^*a*^
pABB32	Mini-RK2 derivative, *cat* (Cm^r^) *klcAp_RA3_-xylE lacO*-MCS-*lacO* (38)
pGBT70	*ori*_SC101_ Km^r^ *trfAp-1*_RK2_-*xylE*, here used as a terminator-probe vector (36)
pPT01	*ori*_SC101_ Km^r^, promoterless *xylE*, promoter-probe vector (40)
pUC18	*ori*_MB1_ *bla* (Ap^r^), cloning vector (97)
RA3	IncU Cm^r^ Sm^r^ Su^r^ (24)
pESB2.88	pUC18 with ′*korA-incC*, PCR fragment amplified on RA3 template with primers 45 and 26 inserted between SphI and SalI restriction sites of pUC18 (RA3 coordinates 6288–7114)
pESB13.76	pPT01 with *incCp*-1-*xylE*, PCR fragment amplified on RA3 template with primers 44 and 24 inserted between PaeI and BamHI restriction sites of pPT01 (RA3 coordinates 6036–6622)
pESB13.77	pPT01 with *incCp-2-xylE*, PCR fragment amplified on RA3 template with primers 45 and 24 inserted between PaeI and BamHI restriction sites of pPT01 (RA3 coordinates 6288–6622)
pESB13.79	pPT01 with *incCp*-4-*xylE*, PCR fragment amplified on RA3 template with primers 44 and 46 inserted between PaeI and BamHI restriction sites of pPT01 (RA3 coordinates 6036–6343)
pESB36	pABB32 derivative with *oriT*_RK2_ *repAp-lacZ lacI*^q^-*tacp-korB*_RK2_, Cm^r^ Km^r^, unstable, RK2 mobilizable vector^*b*^
pESB36.29	pESB36 with the synthetic RA3 partition cassette (*korAp-korA-incC-korB-orf11-mpR-parS*)^*b*^
pESB36.35	pESB36 with the synthetic RA3 WT stability module (*orf02p-orf02-klcAp-klcA-orf04-korCp-korC-kfrC-kfrAp-kfrA-korAp-korA-incC-korB-orf11-mpR-parS*)^*b*^
pESB36.38	pESB36 with the synthetic RA3 Δ*korAp* stability module (*orf02p-orf02-klcAp-klcA-orf04-korCp-korC-kfrC-kfrAp-kfrA-korA-incC-korB-orf11-mpR-parS*)^*b*^
pESB36.40	pESB36 with the synthetic RA3 Δ(*orf02p-orf02*) stability module (′*orf02-klcAp-klcA-orf04-korCp-korC-kfrC-kfrAp-kfrA-korAp-korA-incC-korB-orf11-mpR-parS*)^*b*^
pESB36.41	pESB36 with the synthetic RA3 Δ(*klcAp-klcA*) stability module (*orf02p-orf02-orf04-korCp-korC-kfrC-kfrAp-kfrA-korAp-korA-incC-korB-orf11-mpR-parS*)^*b*^
pESB36.44	pESB36 with the synthetic RA3 Δ(*kfrAp-kfrA*) stability module (*orf02p-orf02-klcAp-klcA-orf04-korCp-korC-kfrC-korAp-korA-incC-korB-orf11-mpR-parS*)^*b*^
pMWB5.15	pGBT70 with *trfAp*-1_RK2_-*repBt*^*c*^-*xylE*, PCR fragment amplified on RA3 template with primers 34 and 35 inserted between KpnI and NcoI sites of pGBT70 (RA3 coordinates 1353–1509)
pMWB5.16	pGBT70 with *trfAp*-1_RK2_-*klcAt-xylE*, annealed oligonucleotides 36 and 37 inserted between KpnI and NcoI sites of pGBT70 (RA3 coordinates 3061–3110)
pMWB5.17	pGBT70 with *trfAp*-1_RK2_-*kfrCt-xylE*, annealed oligonucleotides 38 and 39 inserted between KpnI and NcoI sites of pGBT70 (RA3 coordinates 4771–4820)
pMWB5.18	pGBT70 with *trfAp*-1_RK2_-*kfrAt-xylE*, annealed oligonucleotides 40 and 41 inserted between KpnI and NcoI sites of pGBT70 (RA3 coordinates 5981–6030)
pMWB5.19	pGBT70 with *trfAp*-1_RK2_-*orf11t-xylE*, annealed oligonucleotides 42 and 43 inserted between KpnI and NcoI sites of pGBT70 (RA3 coordinates 8701–8750)

a MCS, multiple-cloning site. ′, truncated ORF (position of the symbol indicates 5′ or 3′ end deletion). A list of primers is presented in Table 4.

b A list of the intermediate constructs and a detailed description of plasmid construction are presented in the supplemental material.

c *repBt*, putative Rho-independent transcriptional terminator downstream of the indicated gene.

**TABLE 4:** Oligonucleotides used in this study

Category and no.	Name	Sequence (5′→3′)^*a*^
Primers used in RT-PCR and 5′RACE		
1	10G	cggaattcGGTGGCCCATTTCGTACGTA
2	orf02pR	cgggatccCGATCACGCTCCCAGGTCAA
3	Orf02F	cagaattcATGATCCACACAGCTAACCG
4	SalOrf02	cgcgtcgacATAGGCCAAATCGGCCTACT
5	klcApL	gcgcatgcGGGAGCGTGATCGTTACGGT
6	klcApR	gcggatccATTGCAGCCATACGGCGAGG
7	EcoklcAL	gcgaattcATGATGCACACAGAACTTAATC
8	SalklcAR	cgcgtcgacCTAGTCTATTGCGGCCAAGA
9	KorCpF	cgcagatctGAAATGGTGCCCCTGGTATG
10	PkorCp	gcggatccCAATCTTCAGCAAACGGCCT
11	korCRA3L	gcgaattcATGATTAGACCTGAAACGCT
12	korCRA3R	cggtcgacTTATGTTCGGTCATGGTTTC
13	KFRCFL	gcaagcttgGAAttCATGACCGAACATAAGGCCGA
14	KFRCFP	aacccggggCCGCTCTAGATCGTCTTCAT
15	prkfrA1	gcggatccgcatgcCTCGCTGATAACCTGGCCCT
16	prkfrA2	gcggatccCTCGCGCACCTGCTCATTTG
17	Ia	cgccaattgagatctgaatccATGACCATGATTAAGCCTG
18	IIb	gcggtcgactacgtaAACGACTCGATAGACTGG
19	korApL	gcgaatccAGTTGACTAAGCCAACAGCG
20	korApR	cgggatCCTTCCTAACCAAAGCCGCTAC
21	korAL	cggaattcATGGCGCTGACCAACGAGGA
22	korAR	cggtcgacGTAGTGGACTTGCCAACGCC
23	IncBF1	cggcagcatgCCCAATAGTTAAGGCCATCG
24	IncCprP	gcggatccGTTCCATATCAGCCATC
25	incCL	cggaatccATGAAAATTGTTACTGTATC
26	incCRA3R	cggtcgacTTTACCACTCATTCAGCCAC
27	korBL	cggaattcATGAGTGGTAAAGGGGCAGA
28	korBGSP2	TCTGCCGTTGTCAGTTCGTC
29	korBR	cggtcgacCGAACAACAACAGCACTCCA
30	OriRA3NF	cggaatccacatgtAGTTAGGGGAAGCCGACGAG
31	OriRA3D	cgcgtcgacacatgtCGATAGCTCTTTGCCATTAAC
32	klcAGSP2	AATCCCGCAAGCTGTGATAC
33	kfrCGSP2	AGCTCCGCTTTTGCCCATTC
	AAP	GGCCACGCGTCGACTAGTACGGGIIGGGIIGGGIIG
	AUAP	GGCCACGCGTCGACTAGTAC
Primers used for cloning		
34	TERREPBF	ccgaattcggtaccACAGGCGGCTAGGTGTAAAG
35	TERREPBR	ccgtcgacacatgtAGGTGGAAGATTAAGCGGGT
36	TERKLCAF	cAGCAACAGAAAGCCCCGGTGATGGCCGGGGCTCTTAAAGGAGGTGCATAGa
37	TERKLCAR	catgtCTATGCACCTCCTTTAAGAGCCCCGGCCATCACCGGGGCTTTCTGTTGCTggtac
38	TERKFRCF	cTGTCACCGACCCCGGCCATTGCGCCGGGTTTTTTTTGTTCGTTTTTCACTa
39	TERKFRCR	catgtAGTGAAAAACGAACAAAAAAAACCCGGCGCAATGGCCGGGGTCGGTGACAggtac
40	TERKFRAF	cTAAAGTGTTATCTTTAAACGGCGGCCAAAATGGCCGCCTGTGATAGGGTGa
41	TERKFRAR	catgtCACCCTATCACAGGCGGCCATTTTGGCCGCCGTTTAAAGATAACACTTTAggtac
42	TERORF11F	cCGAACAAGGGGGCCAAAATCGGCCCCCTTACTTTTGGCGTTTAATCTAGCa
43	TERORF11R	catgtGCTAGATTAAACGCCAAAAGTAAGGGGGCCGTTTTGGCCCCCTTGTTCGggtac
44	IncCprL	cgcgcgcatgcATGGCGCTGACCAACGAG
45	IncCBF1	cggcagcatgcCCAATAGTTAAGGCCATCG
46	IncCAR	gcggattcTTTGGTTTATTAGCCTGT
47	OriTG	acggtcgacacatgtCTGGTTGGCTTGGTTTCATC
48	OriTD	cgGAATTCACATGTTTGCCAAAGGGTTCGTGTAG
49	ODGSN	accatggtCATG
50	Ant5	cgggatccTAGCTGCTGCCAGGATAAAC
51	repAprF	gcgaattcagatcttGCGGGCCTGATCTATTGTTG
52	Oligo1G	aACTAAGCCAACAGCGAAACGCCAACAGAAAAAAGACGCAGCCGACACCAAAGAGTAAgatctCTGCA
53	Oligo1D	GagatcTTACTCTTTGGTGTCGGCTGCGTCTTTTTTCTGTTGGCGTTTCGCTGTTGGCTTAGTt
54	3G	cggaattcCGCGACTCGCTGATAACCTG
55	3D	cgctgcagcaattgggaTCCCTGTATTGTATGTA
56	4D	cgctgcagcaattgggatccctTACAATACAACGGAGTGA
57	7G	cgagaattcGTGCCCCTGGTAT
58	7D	cggGATCcGCATTTGAGTTTGTACGACC
59	8G	cggaattcTCAATtCCGGAGAACTCCGA
60	8DNOWY2	cggtcgacGGGGCACCAaTTgTTTTTCTA
61	9GNOWY	cggaattcctcgagGCAGCCTGGAGCTcAATAAA
62	9D	cgctgcagTCGGAGTTCaCCGGTATTGA
63	10D	cgTTTATTgAGCTCCAGGCTGC
64	ORF02R	gcgtcgactccggAATAGGCCAAATCGGCCTACT
65	1A	gcgaattCGTTaACCATCGACGGTGCCCCGATTG
66	2B	gcgtcgACAGtACTCCAACGGCAACAGCAGC
67	3A	gcgaattcAGcGCTGTTGTTGTTCGCGGCCTATC
68	3B	gcgtcgaccccgGGTGCAATTTTAGCACA
69	6A	gcgaattcagatctATAGGGTGAAGGTCATGGCG
70	6B	gcgtcgaccaattg*ACCCCCA*TCATTCAGCCACCCCCATTT
71	7A	gcgaattcagatctGACACCAAAGAGTAACCCC
Primers used in qPCR analysis^*b*^		
72	orf02F	ATGATCCACACAGCTAACCG
73	orf02R	TAAAACAGACCGACGAGACG
74	klcAF	TGCAATGCCTCGCCATGTAG
75	klcAR	CGTTCGACAGCTCCCACATAAG
76	korCF	GGTGTTGGAGCTGATTAGGC
77	korCR	GAAGGTTCGGGTTCCCTTTC
78	kfrCF	CCTGTCCTGGCTGTTGAGTC
89	kfrCR	TGTACGACCGACCTTTTCCG
80	kfrAF	ATCAGGAGCGTGATGAAGCC
81	kfrAR	TCAACCTCTGAAGCCAAGCC
82	korAF	AACGAGGAATTTGAATCAGCAC
83	korAR	CTTAGGCCGGACTCTTTCAC
84	incCF	ACGAAGACCCTTACCGTATCC
85	incCR	CCCGTTCCATATCAGCCATC
86	korBF1	TCCGTTCAAGCCTTGGCTATC
87	korBR1	GTGCTCGGGTTCTTCAGGTC
88	orf11F	TTGTTGTTCGCGGCCTATCC
89	orf11R	AACGTCGCCTGGTAAAAGCTG
90	CysGF	TTGTCGGCGGTGGTGATGTC
91	CysGR	ATGCGGTGAACTGTGGAATAAACG
92	PArefF7	CCAAGCTGGCTGTCCTCTTC
93	PArefR7	CCGAAGAACTGGCCGAAAAG
94	AgrF	TAGGTGTCGGCAATGGTGTC
95	AgrR	ACGAGGATGTGACTGACGTG
96	RalstFP	GCCTGCACCACCTTGTCTTC
97	RalstRP	TGTGGATGGTGACCTGGATCT
98	PutRefF	TCCGGAGCACTCTCGAATAC
99	PutRefR	CGCAACAGCAGTCTCGTATC
100	EcCysGzF	GCATTAGCGTTTATTCCACAG
101	EcCysGzR	GAGAAGGCTTTCATCAAATGG
102	pEStfAzF	CGATCACCTTCACGTTCTAC
103	pEStfAzR	CGGCCTTCGTGTAATACC

a Restriction enzyme recognition sites or overhangs are underlined, other sequences noncomplementary to the template are shown in lowercase, and an additional Shine-Dalgarno sequence is shown in italic.

b Primers used in qPCR for the reference genes (90 to 99) are as follows: primers 90/91, *cysG* of E. coli; primers 92/93, *rpoD* of P. aminovorans; primers 94/95, *rpoD* of A. tumefaciens; primers 96/97, *gyrB* of C. necator; primers 98/99, *rpoD* of P. putida. Primers used in qPCR for plasmid copy determination (100 to 103) are as follows: primers 100/101, reference chromosomal gene *cysG* of E. coli; primers 102/103, plasmid gene *trfA*.

### Plasmid construction.

The medium-copy-number promoter-probe vector pPT01, based on the pSC101 replicon (40), was used to monitor the promoter activity of the cloned DNA fragments. PCR products were cut with BamHI and PaeI and ligated into pPT01 upstream of the promoterless *xylE* cassette. To test the activity of putative transcriptional terminators, pGBT70 *trfAp-1-xylE* (36), a pPT01 derivative, was used. The point mutation in the −10 box of a very strong *trfAp* of RK2 (*trfAp-1*) lowers its transcriptional activity more than 10-fold, making it suitable for assays of weak transcription termination signals (37). PCR-amplified fragments or double-stranded oligonucleotides corresponding to the putative transcriptional terminators/attenuators were inserted between KpnI and NcoI restriction sites in the test vector to separate *trfAp-1* from the *xylE* cassette. Catechol-2,3-dioxygenase (XylE) activity assays were indicative of functionality of the inserts in modulation of *xylE* transcription.

Previously constructed pABB32 (38), an unstable broad-host-range (BHR) test vector based on the RK2 minireplicon from the IncP-1α incompatibility group, was further modified to extend its use in various bacterial hosts. First, *oriT*_RK2_ was PCR amplified on the RK2 template and cloned as a PscI restriction fragment into the unique NcoI cleavage site of pABB32 to facilitate conjugative mobilization of the obtained pESB29 to chosen species, with the use of the RK2 conjugative system integrated into the E. coli S17-1 genome. Next, the *lacI*^q^ *tacp-korB*_RK2_ cassette was inserted between the BamHI and NruI restriction sites of pESB29 to further downregulate the *trfA* gene expression and to decrease the copy number of the resulting pESB33 construct. Subsequently, the BamHI-PscI restriction fragment of pESB33, carrying the *klcAp*_RA3_-*xylE* cassette, was replaced by the *repAp*_RA3_-*lacZ* transcriptional fusion for easy monitoring of the resulting pESB35 plasmid segregation in an even broader range of hosts (white/blue colonies on L agar with X-Gal). Finally, the kanamycin resistance cassette (*aphA1*) was cloned into the MunI restriction site of pESB35 next to *cat* (Cm^r^) to give pESB36.

The synthetic RA3 stability module was constructed in two pieces (part 1, *orf02p-orf02-klcAp-klcA-orf04-korCp-korC-kfrC-kfrAp-kfrA*; part 2, *korAp-korA-incC-korB-orf11-mpR-parS*) by a multistep procedure of joining restriction and PCR-amplified fragments in the high-copy-number vectors. This strategy, described in detail in the supplemental material, allowed for the construction of various deletion derivatives deprived of particular promoter regions and ORFs. After thorough verification of the DNA sequence, especially at junction points, variants of the RA3 stability module were recloned into the BHR unstable vector pESB36 and used in RT-qPCR assays of transcriptional activities as well as in the plasmid stable maintenance experiments.

### Bacterial transformation.

Competent cells of E. coli were prepared by the standard CaCl_2_ method (87).

### Conjugation procedure.

E. coli strain S17-1 was transformed with pESB36, pESB36.35, and its deletion variants, and such transformants were used as donors in conjugation with the following recipient strains: A. tumefaciens LBA1010R Rif^r^, P. aminovorans JCM7685 Rif^r^, P. putida KT2442 Rif^r^, or C. necator 7MP228r Rif^r^. Cells from the stationary-phase cultures of recipients and donors (grown under antibiotic selection) were washed twice with L broth and resuspended in the initial volume of the medium. Aliquots (100 μl) of donor and recipient were mixed, spotted on an L agar plate, and incubated for approximately 24 h at 28°C. Cells were suspended in 2 ml of L broth, and 100 μl of serial dilutions was plated on L agar with rifampin, X-Gal, and the appropriate selective antibiotic and incubated at 28°C.

### RNA isolation and analysis.

**(i) RT reaction followed by PCR.** An overnight E. coli DH5α(RA3) culture was diluted 1:100 into fresh L broth supplemented with chloramphenicol and propagated with shaking until the optical density at 600 nm (OD_600_) reached 0.4 to 0.6. Two-milliliter samples were mixed with 4 ml of RNAprotect Bacteria reagent (Qiagen). Total RNA was isolated using the RNeasy minikit (Qiagen) according to the manufacturer’s instruction and treated with the Turbo DNase kit (Ambion) to remove DNA contamination. The control PCRs were conducted on purified RNA to ensure lack of a DNA template. The RNA concentration was estimated using the NanoDrop ND-1000 spectrophotometer (Thermo Scientific). The integrity and overall quality of RNA preparation were assessed by native agarose gel electrophoresis (87) (see Fig. S1 in the supplemental material). cDNA synthesis and purification were performed with the 5′RACE System for Rapid Amplification of cDNA Ends, version 2.0 (Invitrogen). Briefly, 4 μg of total RNA was used per reaction with SuperScript II reverse transcriptase (RT) and primer 8, 14, or 29 (Table 4). The bulk of the RNA was removed with a mixture of RNases T1 and H. cDNA was then used as a template for PCRs with appropriate pairs of primers. PCR products were analyzed by agarose gel electrophoresis, and gels were stained with ethidium bromide and photographed.

**(ii) RT-qPCR analysis.** Three independent cultures of each analyzed strain, i.e., E. coli EC1250, A. tumefaciens LBA1010R Rif^r^, P. aminovorans JCM7685 Rif^r^, P. putida KT2442 Rif^r^, or C. necator 7MP228r Rif^r^ carrying pESB36.35 (WT RA3 stability module) or its derivatives (pESB36.29, pESB36.38, pESB36.40, pESB36.41, or pESB36.44), were grown until the stationary phase of growth at 37°C (E. coli) or 28°C (other strains) in L broth supplemented with appropriate antibiotics. Cultures were diluted 1:100 into fresh L broth with antibiotic selection and propagated to an OD_600_ of 0.4 to 0.6. Total RNA was isolated from logarithmic-phase cultures and prepared as described above. The first-strand cDNA was synthesized with the TranScriba kit (A&A Biotechnology) using 1 to 3 μg of total RNA per reaction with random hexamer primers. RT-qPCRs were performed in the LightCycler 480 system (Roche Life Sciences, Penzberg, Germany) using Hot FIREPol EvaGreen qPCR Mix Plus (Solis Biodyne) according to the manufacturer’s instructions. The reactions were carried out with 1 μl of 6-times-diluted cDNA in a total volume of 19 μl, and three technical replicates were done for each gene/primer combination. Primers used to amplify the reference and target genes were checked for specificity and efficiency (only primers with amplification factor between 1.95 and 2 were used). Target gene expression was normalized to the reference gene, i.e., *cysG* (E. coli) (39), *rpoD* (P. putida, P. aminovorans, and A. tumefaciens) (89), or *gyrB* (C. necator) (89, 90), and calculated using the Pfaffl method (91). The mean values with standard deviations from three independent biological samples for each strain/plasmid combination were reported.

**(iii) 5′RACE method.** The 5′ rapid amplification of cDNA ends (5′RACE) technique (35) was done using the 5′RACE System for Rapid Amplification of cDNA Ends, version 2.0 (Invitrogen), as outlined in the manufacturer’s protocol.

**(a) Identification of multiple transcripts for ORFs in the RA3 stability module.** Total RNA from E. coli DH5α(RA3) and cDNAs (with a gene-specific [GSP1] primer [8, 14, or 29]) were prepared as described above for the RT-PCR technique. Next, homopolymeric tails were added to the 3′ ends of the cDNAs using terminal deoxynucleotidyl transferase (TdT) and dCTP. The tailed cDNA was PCR amplified with the abridged anchor primer (AAP), containing a run of Gs at the 3′ end, and an appropriate nested (GSP2) primer (32, 33, or 26). To increase the sensitivity of detection, the obtained PCR products were then used as templates in subsequent reactions with appropriate pairs of nested primers homologous to the studied sequence (3/4, 7/8, 11/12, 13/14, 13/33, 17/18, 21/22, and 25/26). Following PCRs, products were analyzed by agarose gel electrophoresis, and gels were stained with ethidium bromide and photographed.

**(b) Identification of transcription start sites of *incC*.** Total RNA isolated from E. coli DH5α(RA3) was primed with primer 29 and used for cDNA synthesis. cDNAs were 3′ dC tailed as described above and used as templates in PCRs with primers AAP and 26 (Fig. 2J). The primary PCR products then were reamplified using the abridged universal amplification primer (AUAP), which is identical to the 5′ part of AAP, paired with primer 24 (Fig. 2A). Following amplification, 5′RACE products were separated by agarose gel electrophoresis, extracted from the gel using the Gel-out kit (A&A Biotechnology), and sequenced.

### Determination of catechol 2,3-dioxygenase (XylE) activity.

Overnight cultures of E. coli C600K transformants of the promoter-probe (pPT01) or terminator-probe (pGBT70) vectors and their derivatives were diluted 1:50 into fresh L broth supplemented with an appropriate antibiotic and grown to an OD_600_ of 0.5 to 0.8. Catechol 2,3-dioxygenase activity was assayed spectrophotometrically in cleared supernatants of sonicated cells as described previously (92). The reaction was initiated by addition of catechol (final concentration, 15 μM). One unit of catechol 2,3-oxygenase activity is defined as the amount of enzyme needed to convert 1 μmol of catechol to 2-hydroxymuconic semialdehyde per mg of protein in 1 min. Protein concentration was determined using the Bradford method (93). Experiments were performed in triplicate, and the mean values with standard deviations were reported.

### Determination of plasmid stability.

Three independent cultures of each analyzed strain, i.e., E. coli EC1250, A. tumefaciens LBA1010R Rif^r^, P. aminovorans JCM7685 Rif^r^, P. putida KT2442 Rif^r^, or C. necator 7MP228r Rif^r^ carrying pESB36, pESB36.35, pESB36.29, pESB36.38, pESB36.40 pESB36.41, or pESB36.44, were cultivated until the stationary phase of growth at 37°C (E. coli) or 28°C (other strains) in L broth supplemented with an appropriate antibiotic. Subsequently, the cultures were diluted 10^5^-fold into fresh L broth (without antibiotic selection) and grown for up to 20 generations as described above. The procedure was then repeated twice (after every 20 generations) until at least 60 generations of growth without selection. In parallel, with every passage step, 100-μl aliquots of the 10^−6^ dilution of cultures were plated on L agar supplemented with 40 μg ml^−1^ X-Gal and incubated at 28°C or 37°C to obtain approximately 100 to 350 colonies. Plasmid retention was calculated as a percentage of blue colonies. The plasmid loss rate (LR) per generation (%) was calculated using the formula ($1-\sqrt[{n}]{\frac{F_{f}}{F_{i}}})\times 100$, where *n* is the number of generations, *F_i_* is the fraction of cells containing plasmid at the initial time point, and *F_f_* is the fraction of cells containing plasmid at the final time point. The stability index (SI) for each construct was calculated as the ratio of the rate of loss of pESB36 to the rate of loss of the vector with the RA3 stability module variant in each host (94).

### Plasmid copy number.

The copy number of pESB36 and pESB36.35 in various hosts was estimated by real-time qPCR. E. coli EC1250, A. tumefaciens LBA1010R Rif^r^, P. aminovorans JCM7685 Rif^r^, P. putida KT2442 Rif^r^, and C. necator 7MP228r Rif^r^ carrying pESB36 or pESB36.35 were grown on L broth with a selective antibiotic to the stationary phase. Total bacterial DNA was extracted using a modification of the method of Chen and Kuo (95, 96). Briefly, cells were collected from 5 ml of the cultures by centrifugation and suspended in 400 μl of TESO lysis buffer (40 mM Tris-HCl [pH 8.0], 1 mM EDTA [pH 8.0], 1% [wt/vol] SDS, 20 mM sodium acetate) and 132 μl of 5 M NaCl. The samples were vortexed for 1 min and then centrifuged at 4°C for 10 min at 16,000 × *g*. One volume of phenol-chloroform (1:1 [vol/vol]) was added to the supernatants, and samples were vortexed for approximately 1 min and centrifuged as before. The extraction procedure was repeated for the upper phase, with an equal volume of chloroform. Subsequently, the aqueous fraction was collected, and DNA was precipitated with 2 volumes of 98% ethanol (87). Fifty and 100 nanograms of each DNA template were used in qPCRs with 5× Hot FIREPol EvaGreen qPCR Mix Plus (Solis Biodyne), and reactions were carried out according to the manufacturer’s instructions in the LightCycler 480 Instrument II (Roche). Single-copy genes on the chromosomes were chosen as the reference genes: *cysG* for E. coli, *rpoD* for P. putida, P. aminovorans, and A. tumefaciens, and *gyrB* for C. necator. *trfA* was used as a plasmid target for pESB36 and pESB36.35 (pairs of primers are listed in Table 4). The primers were checked for specificity and efficiency (only primers with an amplification factor between 1.95 and 2 were used). All qPCRs were done in triplicates. The PCN, defined as the number of plasmid amplicons relatively to the number of chromosome amplicons, was calculated considering the amplification efficiencies of the primers used (95, 96). The average results of at least four biological replicates with standard deviation were reported.

## Supplementary Material

Supplemental file 1
